# Changes in the Metabolism of GD2-Specific Murine CAR-T Cells After Co-Culturing with Melanoma

**DOI:** 10.3390/ijms27115093

**Published:** 2026-06-04

**Authors:** Aleksey Bulygin, Julia Philippova, Nikita Basov, Ekaterina Butikova, Maria Sotnikova, Yulia Sotnikova, Yuriy Patrushev, Artem Rogachev, Evgeniy Gaisler, Andrey Pokrovsky, Hiroshi Shiku, Sergey Sennikov

**Affiliations:** 1 Laboratory of Molecular Immunology, Federal State Budgetary Scientific Institution Research Institute of Fundamental and Clinical Immunology, 630099 Novosibirsk, Russia; aleksej.bulygin95@mail.ru (A.B.); airyuka@mail.ru (J.P.); shiku@clin.medic.mie-u.ac.jp (H.S.); 2 N.N. Vorozhtsov Novosibirsk Institute of Organic Chemistry, Siberian Branch of the Russian Academy of Sciences, 630090 Novosibirsk, Russia; nikita.v.basov@gmail.com (N.B.); m.sotnikova1@g.nsu.ru (M.S.); sotnikova@nioch.nsc.ru (Y.S.); i.patrushev@g.nsu.ru (Y.P.); rogachev@nioch.nsc.ru (A.R.); 3 Novosibirsk State University, 630090 Novosibirsk, Russia; katabutikova@gmail.com (E.B.); evgeniy.gaysler@mail.ru (E.G.); agpok@inbox.ru (A.P.); 4 Research Institute of Clinical and Experimental Lymphology—Branch of the Institute of Cytology and Genetics, Siberian Branch of the Russian Academy of Sciences, 630090 Novosibirsk, Russia; 5 Boreskov Institute of Catalysis, Siberian Branch of the Russian Academy of Sciences, 630090 Novosibirsk, Russia; 6 Federal State Autonomous Educational Institution of Higher Education I.M. Sechenov, First Moscow State Medical University of the Ministry of Health of the Russian Federation, 119435 Moscow, Russia

**Keywords:** GD2-specific CAR-T cells, B78-21 melanoma, transduction, co-culturing, metabolic profiling, LC-MS/MS, ATP production

## Abstract

Immunotherapy with the use of induced CAR-T cells is an effective method of cancer suppression in close to in vivo conditions. The question of the nature of metabolism of CAR-T cells in the conditions of fighting not against single cancer cells, but against tumor cells, remains relevant. Our studies have shown the metabolomic profile of mouse GD2-specific CAR-T cells upon contact with B78-21 melanoma tumor cells and upon exposure to B-21 melanoma 3D spheroid structures.

## 1. Introduction

Adoptive cell therapy (ACT) represents a promising treatment option that harnesses the immune system to target solid tumors, which often exhibit immunosuppressive microenvironments [1,2]. Currently, ACT employs various approaches, including the use of chimeric antigen receptor (CAR) T cells, which are genetically engineered T lymphocytes expressing a synthetic receptor that redirects their specificity toward tumor-associated antigens [3,4]. Unlike conventional T cells that rely on the native T-cell receptor (TCR) to recognize peptide antigens presented by major histocompatibility complex (MHC) molecules, CAR-T cells bind directly to native surface antigens in an MHC-independent manner, enabling robust activation upon antigen encounter. However, CAR-T cells can exhibit significant toxicity against normal tissues expressing the target antigen at low levels [5,6]. Therefore, achieving a balance between high efficacy and low toxicity through modulation of signaling pathways in adoptive cell therapy remains a key priority in clinical practice.

To date, it is known that tumor cells have a negative impact not only on the effector function of T cells but also on their metabolic function in general [7,8]. For example, tumor cells have a particular type of metabolism described as the Warburg effect [9], which leads to the release of large amounts of lactic acid with a subsequent decrease in pH in the medium has multiple inhibitory effects on the activity and function of tumor-infiltrating lymphocytes (TILs), especially cytotoxic T lymphocytes (CTLs). CAR-T cells have similar metabolic working principles as cytotoxic T lymphocytes, which is expressed in the relationship between T cell receptor activation and metabolic reprogramming [10]. At the same time, it is known that T-cell receptor and costimulatory factors CD28 and 4-1BB, when triggering T-cell pathway activation of T-cell immune function, also trigger transcription factors (mTORC1, HIF-1a, etc.) that regulate the directionality of cell metabolism [11,12]. This, in turn, contributes to the provision of energy and components that are essential for cytokine production and maintenance of immune function of cells in the tumor microenvironment [11]. Therefore, to date, many researchers have sought alternative approaches to regulate the metabolism of T cells to enhance their ability to fight tumor cells [13,14]. To address this, our study aimed to investigate the metabolic profile of GD2-specific CAR-T cells working in response to tumor cells.

In this study, our focus is on unraveling the metabolic dynamics of three distinct approaches to engineer TCR (T cell receptor) within the tumor microenvironment. T-lymphocyte populations are transduced with a unique retroviral vector, encoding GD2-specific CAR-T cells in mice. The resulting GD2-specific CAR-T cells were co-cultured with B-78-21 tumor cells. To understand the metabolic dynamics in the interaction between T cells and melanoma, we used 3D structures of tumor cells in vitro. To understand which groups of metabolites are involved in regulating CAR-T-cell metabolism, we used GD2-specific CAR-T cells co-cultured with tumor cells in vitro. Our results lay the foundation for a more detailed study of the intermolecular interactions of metabolic and immunological signaling pathways under different external microenvironmental factors. These data will help to adjust the future application of CAR-T cells in cancer immunotherapy.

## 2. Results

**Figure 1:**
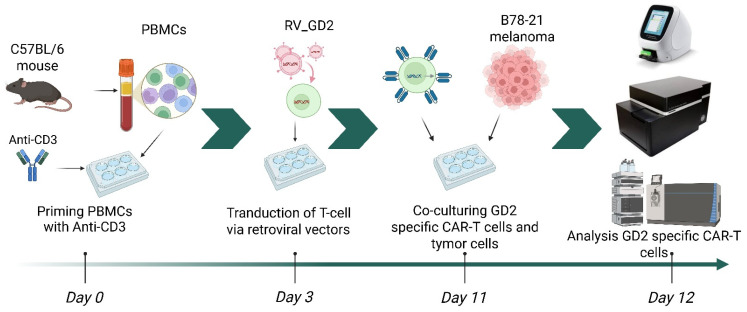
Experimental design to evaluate the metabolism of mouse GD2-specific CAR-T cells co-cultured with GD2-positive B78-21 melanoma tumor cells. A more detailed description of the steps for obtaining CAR-T cells and co-culturing them with tumors is provided in Section 4.

To determine the metabolic dynamics of GD2-specific CAR-T cells upon contact with tumors, we transduced isolated CD3+ T cells from splenocytes of C57Bl/6 mice with a retrovirus encoding the anti-GD2 CAR. The resulting GD2-specific CAR-T cells and non-transduced T cells (RV-T) were then co-cultured with B78-21 melanoma tumor cells (Figure 1). Following Liquid Chromatography-tandem Mass Spectrometry (LC-MS) analysis of metabolites, we decided to test the metabolic activity of GD2-specific CAR-T cells using a 3D spheroid of the B78-21 melanoma cell line and analyze the metabolic activity using Seahorse and fluorescence microscopy (Figure 1).

### 2.1. CD8+ and CD44+CD62L− Cells Make up the Majority of the CD3+ CAR T Cell Population

**Figure 2:**
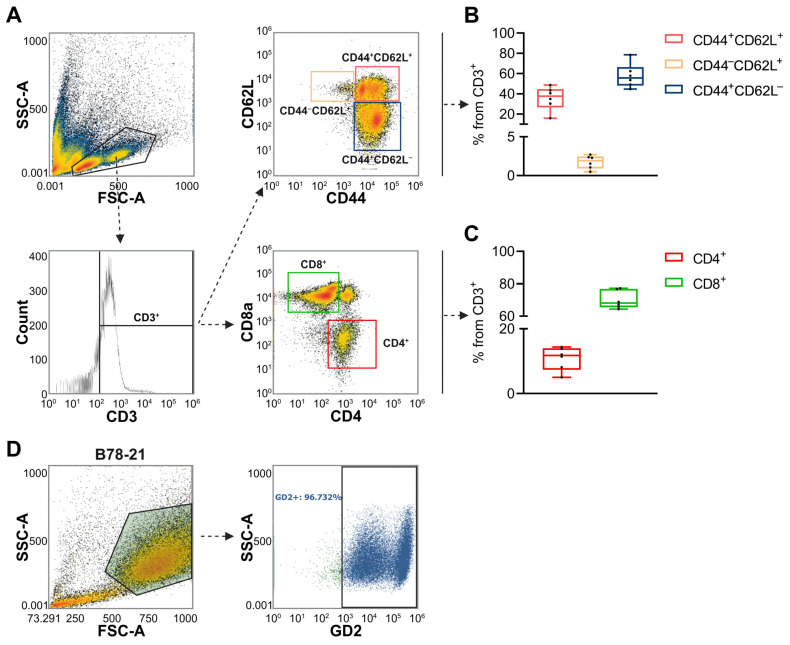
Mouse CD3+ CAR-T cell subset analysis and GD2 antigen expression by the B78-21 melanoma cell line. (**A**)—Gating strategy for mouse CAR-T cell subset analysis. (**B**)—Boxplots showing the distribution of the resulting GD-specific GAR T cells (n = 6). (**C**)—Boxplots showing the subset composition of CD4+ and CD8+ T cells (n = 6). Data are presented as mean ± SEM. (**D**)—GD2 antigen expression by the B78-21 melanoma cell line (96.73%).

Prior to conducting the metabolomic analysis, the subpopulation composition of GD2-specific CAR-T cells was assessed using flow cytometry. A study of CAR-T cell subpopulations revealed that CD8+ T cells predominated (70.07 ± 2.28%) compared to CD4+ T cells (10.81 ± 1.47%) (Figure 2C). Depending on the expression of CD44 and CD62L markers, the T cell population of mice can be divided into three main subpopulations: CD44+CD62L+ central memory T cells (TCM), CD44+CD62L− effector memory T cells (TEM), and CD44−CD62L+ naive T cells (TN) [15,16]. Accordingly, the CAR-T cells were dominated by the TEM population (57.68 ± 4.86%), while TCM (35.59 ± 4.72%) and less than 2% TN (1.75 ± 0.35%) were also detected (Figure 2B).

### 2.2. Retrovirus Transduction of GD2-Specific TCR Enhances T Cell Proliferation and Modifies the Metabolic Profile of T Cells

**Figure 3:**
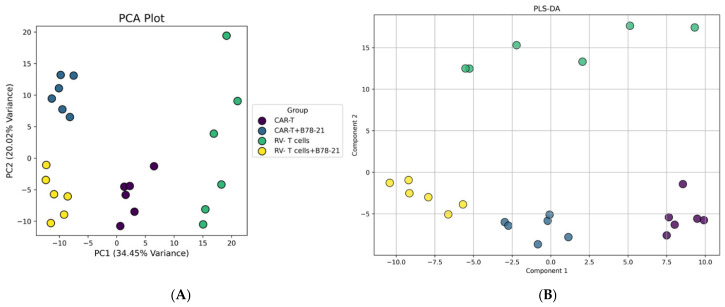
Principal component analysis (PCA) score plot (**A**) and Partial Least-Squares Discriminant Analysis (PLS-DA) score plot (**B**) of GD2-specific CAR-T cells and non-transduced T cells before and after co-culturing with tumor B78-21 melanoma (n = 6). The PCA score plots and the PLS-DA score plot showed that samples CAR-T cells, RV-T cells, CAR-T + B78-21, and RV-T cells + B78-21 were clustered separately.

Principal component analysis (PCA) revealed a distinct separation between the studied groups (Figure 3A). The co-culture of CAR-T and RV-T cells with tumor cells had a significant impact on the metabolomic profile of cells, both between the groups and when comparing CAR-T and RV-T cells without co-culture with tumors (Figure 3A). Further PLS-DA analysis confirmed the formation of four distinct clusters, including CAR-T, CAR-T + B78-21, and RV-T cells + B78-21, which share similar features in component 2 (see Figure 3B). Consequently, it can be deduced that both transduction and tumor factors exert a significant influence on the comprehensive metabolic profile of GD2 CAR-T cells.

### 2.3. CAR-T Cells Have Been Observed to Demonstrate Significant Metabolic Reprogramming and Alterations in Lipids in Response to Co-Culturing with Melanoma Cells

We then analyzed the data, identifying 242 metabolites with significantly different values (Supplementary S1). The control group demonstrated elevated levels of sphingolipids (ceramide) and molecules implicated in the biosynthesis of arginine, ornithine, citrulline, valine, isoleucine, and leucine (Supplementary S1). Furthermore, RV-T cells co-cultured with melanoma exhibited a shift in their metabolite profile towards the biosynthesis of glutathione, ascorbate, adenosine, and one-carbon molecules (Supplementary S1). The CAR-T cell group that was not co-cultured with tumors exhibited elevated levels of metabolites implicated in the biosynthesis of arginine, ornithine, citrulline, valine, isoleucine, and leucine (Supplementary S1). The co-culture of the B78-21 melanoma cell line with the GD2-specific CAR-T cells resulted in alterations to their metabolomic profile, with changes observed in the metabolism of creatine (creatine, creatinine, guanidinoacetate) and essential amino acids (glutamine, glutamate, proline, histidine) (Supplementary S1). Overall, the transduction of cells with GD2-specific antigens induces profound reprogramming, with the formation of the effector phenotype in GD2-specific CAR-T cells increasing their dependence on glycolysis, glutaminolysis, and antioxidant defense, while non-transduced T cells maintain classical metabolic principles.

**Figure 4:**
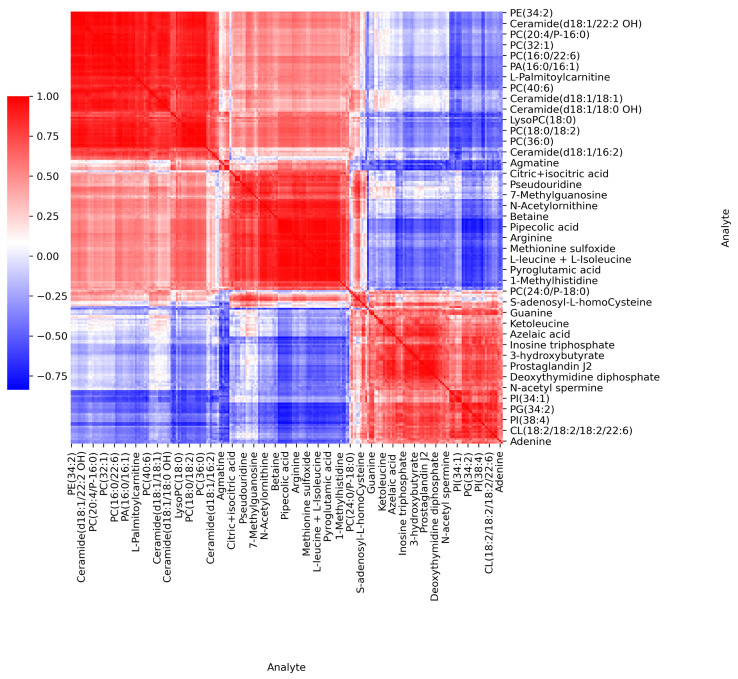
Heatmap of the correlation matrix between metabolites. The matrix was calculated based on metabolite data, and the heatmap visualizes the relationships between them. Colors indicate the strength and direction of correlations. Dark blue indicates a negative correlation between two variables, where an increase in one variable corresponds to a decrease in the other. White indicates zero or very weak correlation, meaning the variables are either uncorrelated or only minimally correlated. Red indicates a positive correlation between two variables, where an increase in one variable corresponds to an increase in the other. The darker the red, the stronger the positive correlation.

Based on the obtained data, we created a correlation matrix that demonstrates distinct patterns of lipid metabolism regulation. The figure shows a high content of lipids, predominantly ceramides, as well as enzymes such as serine palmitoyltransferase and ceramide synthase, and the adjacent phosphatidylcholine subcluster (e.g., PC (32:1), PC (34:2), and PC (36:0)) in GD2-specific CAR-T cells (Figure 4). The results obtained demonstrate strong positive correlations (r > 0.8), thus indicating a unified change in glycerophospholipid regulation (Figure 4). Moreover, the figure demonstrates a correlation with antagonistic relationships. The central diagonal of the graph demonstrates moderate to strong negative correlations (r < −0.5) between sphingolipid clusters and certain glycerophospholipids, including phosphatidylethanolamines (e.g., PE (34:2)) and phosphatidylinositols (e.g., PI (34:1), PI (38:4)) (Figure 4). Furthermore, bioactive metabolites, including arginine sulfoxide, methionine sulfoxide, and S-adenosyl-L-homocysteine (a key player in one-carbon metabolism and methylation), have been observed to demonstrate a negative correlation with ketone-related lipids, such as 3-hydroxybutyrate (see Figure 4). In smaller clusters, mixed patterns of correlations arise associated with amino acid-derived lipids (e.g., leucine + isoleucine, arginine sulfoxide), with moderate positive associations with PC (r ≈ 0.4–0.6), linking amino acid catabolism to phospholipid synthesis via acetyl-CoA intermediates. Finally, negative correlations are also observed, predominantly in the lower right quadrant, where PI and CL (cardiolipins, e.g., CL (18:2/18:2/18:2/6)) anticorrelate with upstream sphingolipids (r < −0.6), thereby providing further support for the hypothesis that the metabolic profile of T cells is highly variable upon transduction and co-culture with tumor cells.

### 2.4. The Resulting gd2-Specific CAR T Cells Show a Multiple Increase in Metabolic Activity upon Contact with Tumors

**Figure 5:**
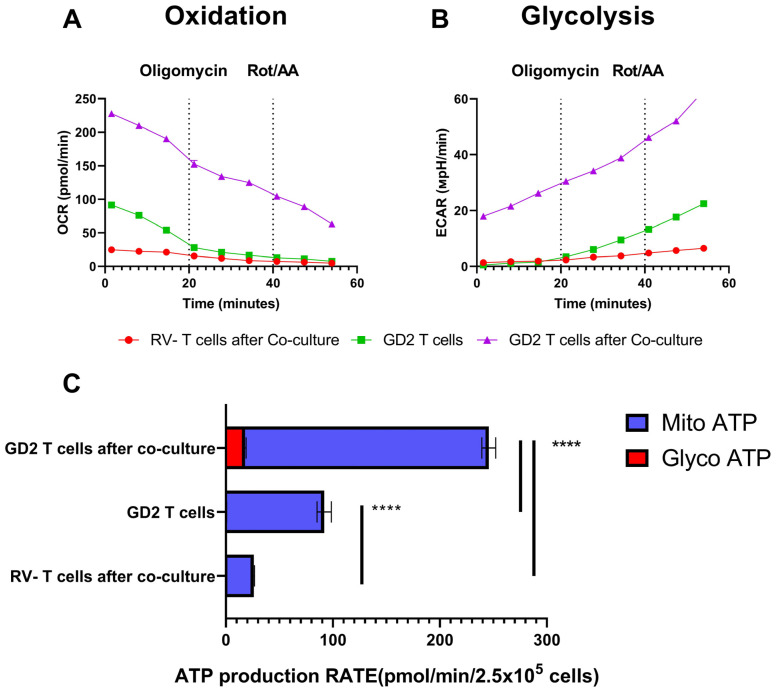
Determination of ATP production in GD2-specific CAR-T cells after co-culture with B78-21 melanoma spheroids (24 h) (n = 6). (**A**)—OCR kinetic profile of GD2-specific CAR-T cells and control cells co-cultured with spheroids. (**B**)—ECAR kinetic profile of GD2-specific CAR-T cells and control cells co-cultured with spheroids. (**C**)—Analysis of metabolic profile of GD-2-specific CAR-T cells and control cells co-cultured with spheroids, without, and in the control group. Dashed lines indicate the addition of oligomycin (1.5 μM) and rotenone-antimycin A (0.5 μM) inhibitors. Data are expressed as the mean ± SEM. The bar indicates the significance of differences between the determined groups. Paired two-tailed Student’s *t*-test was used for data analysis, ****—*p* < 0.0001.

When we assessed metabolic activity by analyzing the difference in mitochondrial and glycolytic ATP production in GD2-specific CAR-T cells in contact with and away from tumors, we revealed the following. The basal level of OCR was increased several-fold in GD2-specific CAR-T cells that were co-cultured with spheroids compared to the control (RV-T cells after co-culture: 226 pmol/min; GD2 T cells: 92 pmol/min; GD2 T cells after co-culture: 24 pmol/min) (Figure 5A). ECAR analysis showed an increased basal level of ECAR several-fold higher in GD2-specific CAR-T cells co-cultured with spheroids, as well as a threefold increase in glycolytic ATP production after the addition of oligomycin and rotenone/antimycin A inhibitors (Figure 5B). To determine the metabolic profile of GD2-specific CAR-T cells, the ratio of basal mitochondrial and glycolytic ATP production was calculated. The results showed that oxidative phosphorylation prevailed over glycolysis in all groups (Figure 5C). Furthermore, the overall level of ATP production was significantly higher in GD2-specific CAR-T cells co-cultured with spheroids than in control GD2 CAR-T cells and non-transduced T cells (Figure 5C). Finally, the initial level of ATP production in GD2-specific CAR-T cells that did not come into contact with tumors was significantly higher than that in RV T cells co-cultured with tumor cell spheroids (Figure 5C).

**Figure 6:**
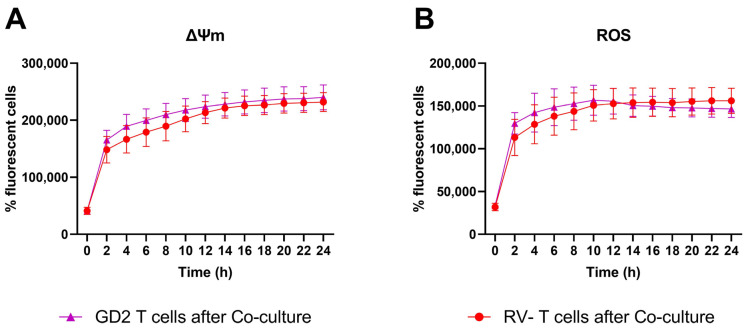
Determination of mitochondrial membrane potential and reactive oxygen species in GD2-specific CAR-T cells with three-dimensional culture of B78-21 melanoma tumor cells over a period of 24 h (n = 6). (**A**)—mitochondrial membrane potential (Δψm) in GD2-specific CAR-T cells and non-transduced T cells (RV-) with three-dimensional culture of B78-21 melanoma. (**B**)—reactive oxygen species (ROS) in GD2-specific CAR-T cells and non-transduced T cells (RV-) with three-dimensional culture of B78-21 melanoma. Data are presented as mean ± SEM. Standard two-way analysis of variance with Tukey’s multiple comparison test was performed.

Analysis of the mitochondrial membrane potential (Δψm) and the levels of reactive oxygen species (ROS) in GD2-specific CAR-T cells that were co-cultured with melanoma tumor cell spheroids for 24 h revealed the following: From 2 h of culture onwards, both GD2 CAR-T cells and non-transduced T cells exhibited an increase in the proportion of cells with high levels of both ROS and Δψm at all-time points during the experiment. ROS and Δψm levels remained consistent in both groups throughout the observation period (see Figure 6A,B). Overall, these data demonstrate that the altered metabolomic profile of both GD2-specific CAR-T cells and the control group upon contact with tumors results in sufficiently high mitochondrial activity in the presence of reactive oxygen species, contributing to tumor suppression and T-cell survival in these microenvironmental conditions.

## 3. Discussion

Metabolomic analyses of mouse GD2-specific CAR-T cells co-cultured with the B78-21 melanoma tumor cell line reveal coordinated upregulation of lipids to support membrane biogenesis and signaling during T-cell expansion. Negative interclass correlations between sphingolipids such as phosphatidylinositol and cardiolipin resonate with depleted urea cycle and amino acid clusters in tumor-co-cultured CAR-T cells. This highlights potential trade-offs, whereby ceramide accumulation promotes stress responses at the expense of mitochondrial integrity [17,18]. Multivariate metabolomic profiling reveals activation-driven changes (transduction), as well as increased glycolysis and amino acid utilization in response to tumor-derived signals. Furthermore, Seahorse data confirm the hypothesis of strong metabolic activation, which increased several times above glycolytic and mitochondrial ATP levels in CAR-T cells following co-culture with tumors [19]. Despite high levels of reactive oxygen species in cells that can lead to apoptosis, strong mitochondrial activity was observed in the CAR-T cells [20]. Together with those received earlier studies that observed increased TIM3+ with low PD-1 expression in CD2-specific CAR-T cells, this has a logical explanation, indicating an activated, but not depleted, CAR-T phenotype in the tumor microenvironment [4]. Additionally, this is consistent with heatmap and correlation matrix data regarding the impact of tumors and hyperactivity on metabolic reprogramming in CAR-T cells [21,22]. The tissue environment in malignant diseases is known to be a fierce confrontation between infiltrating lymphocytes and tumor cells, which is expressed in the struggle for nutrients [23]. For instance, when naïve T cells are activated to become effector T lymphocytes, they reprogram their metabolism towards glycolysis to maintain cellular bioenergetics in the tumor environment [7]. This is consistent with our previous data on the functional activity of GD2-specific CAR-T cells, which showed that GD2-specific CAR-T cells had cytotoxic activity against the targeted antigen [4,24]. Furthermore, cytotoxicity marker analysis [4] also supports our assertion that GD2-specific CAR-T cells, upon contact with tumors, enhance their metabolic capabilities to suppress the tumor environment. In our case, we observe a strong influence of lipid metabolism and amino acid biosynthesis. This is usually characterized by the rapid adaptation of cells to the tumor environment, allowing oxidative phosphorylation and the maintenance of amino acid metabolism to produce cytokines to fight tumors [17,25,26]. However, we also observe increased glycolytic ATP production and high concentrations of branched-chain amino acids (BCAAs), ketone bodies, and glycolytic endpoints (red clusters), reflecting a shift towards catabolic flexibility to support T-cell proliferation and tumor killing [21,27].

Taken together, these metabolomic results suggest that CAR-T cell therapy induces profound metabolic rewiring, shifting CAR-T cells from a quiescent, low-energy state with diffuse correlations and minimal pathway activity to dynamic hyperactivation upon tumor contact. This hyperactivation is fueled by glycolytic flux, glutaminositol, and lipid co-regulation, which can contribute to exhaustion in solid tumor microenvironments such as melanoma [28]. This assertion is supported by previous studies that observed inhibition of tumor growth and an increase in overall survival of tumor-bearing mice to 85 days [4]. The partial overlap between PCA and PLS-DA also suggests the presence of common adaptive mechanisms among different T cell types, including increased antioxidant levels to counteract oxidative stress. Meanwhile, heatmap and correlation patterns reveal vulnerabilities such as BCAA depletion and antagonistic lipid dynamics that could explain limited persistence in vivo [13,29]. These results are consistent with the finding that metabolic reprogramming is a hallmark of effective immunotherapy, but they also identify potential therapeutic targets, such as modulation of sphingolipid pathways or ketone supplementation, to enhance the efficacy of CAR-T cells against resistant tumors [30,31]. Future studies integrating metabolomic and transcriptomic analyses of human CAR-T cells may reveal regulatory nodes involved in shaping the T cell effector phenotype, paving the way for metabolically optimized cell therapies.

## 4. Materials and Methods

### 4.1. Retroviral Particles

**Figure 7:**
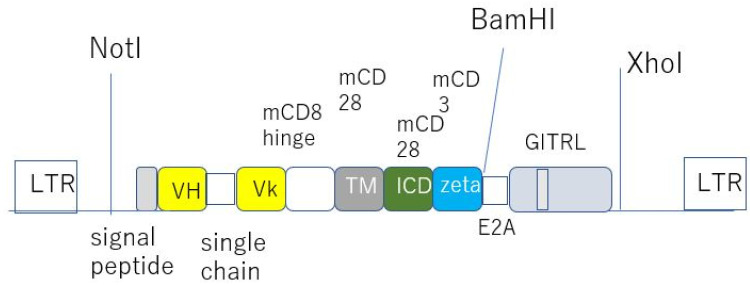
Schematic representation of the retroviral plasmid inserts encoding GD2-specific CAR-T cells.

A retroviral vector encoding a GD2-specific chimeric mouse T cell receptor was developed and provided by the Mie University Graduate School of Medicine, Japan, under the direction of Professor H. Shiku (Figure 7).

### 4.2. Lab Animals

C57Bl/6 mice aged 8–10 weeks were used in the study. The animals were maintained under natural light and unlimited access to food and water at the vivarium of the Research Institute of Fundamental and Clinical Immunology at a meeting on 30 May 2022 [protocol #139]. All animal experiments were pre-approved by the local ethics committee of the RIFCI, adhering to humane principles in accordance with European Community Directive (86/609/EEC). Mice were sacrificed by cervical dislocation under light ether anesthesia.

### 4.3. Isolation of Organs and Splenocytes

The mice’s abdominal cavity was opened using sterile instruments, after which the spleen was removed and transferred to a Petri dish containing an RPMI-1640 liquid nutrient medium (Biolot, Saint-Petersburg, Russia). Splenocytes were obtained by flushing the spleen stroma with a syringe. The resulting cell mass in the medium was collected in test tubes and centrifuged at 1500 rpm for 10 min. To remove red blood cells and spleen stroma, the splenocyte suspension was pre-diluted with RPMI-1640 medium and then centrifuged at 1500 rpm for 10 min in a Ficoll-Urografin density gradient (PanEco, Moscow, Russia). The formed interphase ring of mononuclear cells (MNCs) was then collected, and the cells were purified from the Ficoll-Urografin by two successive washes in phosphate-buffered saline (PBS) (Biolot, Russia), followed by centrifugation at 1500 rpm for 10 min.

### 4.4. Magnetic Separation of Mouse CD3 Lymphocytes

To isolate CD3 lymphocytes from the total cell suspension, magnetic cell separation was performed using a cocktail of biotin-conjugated monoclonal antibodies and streptavidin-conjugated magnetic nanoparticles (BioLegend, San-Diego, CA, USA). The resulting mononuclear cell (MNC) pellet was supplemented with 1 mL of a phosphate-buffered saline (PBS)-based magnetic buffer solution containing 2 mM ethylenediaminetetraacetic acid (EDTA) and 0.5% bovine serum albumin (BSA) (Roche, Rotkreuz, Switzerland), and then centrifuged at 1500 rpm for 10 min. The cells were then separated using a magnetic buffer and a MojoSort™ magnet (BioLegend, USA) in a cold environment, according to the manufacturer’s instructions.

### 4.5. Activation of Mouse CD3 Lymphocytes

The day before the mouse CD3 lymphocytes were isolated, a 6-well plate was prepared by adding 2 μg of mouse anti-CD3ε antibody and 5 μg of mouse anti-CD28 antibody (both from Cloud Clone Corp., Wuhan, China) to each well in 2 mL of PBS solution. The plate was then incubated overnight at 4 °C to allow the antibodies to absorb. The next day, the well was washed with 1 mL of PBS solution and 1.5 × 10^6^ CD3 lymphocytes, obtained after magnetic separation, were seeded in 5 mL of RPMI-1640 medium supplemented with 10% FCS (HyClone, Logan, UT, USA), 2 mM L-glutamine (Biolot, Saint-Petersburg, Russia), 5 × 10^−5^ mM mercaptoethanol (Sigma, St. Louis, MO, USA), 25 mM HEPES (Biolot, Saint-Petersburg, Russia), 80 μg/mL gentamicin (KRKA, Novo mesto, Slovenia), 100 μg/mL ampicillin (Sintez, Kurgan, Russia) (hereafter referred to as ‘full media RPMI-1640′), 60 U/mL hIL-2 (Roncoleukin, Novosibirsk, Russia) and 10 ng/mL IL-7 (BioLegend, San Diego, CA, USA). The plate was then incubated for two days at 37 °C and 5% CO_2_.

### 4.6. Transduction of Mouse T Lymphocytes Using a Retroviral Vector

Retroviral solutions were thawed in a water bath and diluted fourfold with PBS containing 2% human serum albumin (Mikrogen, Moscow, Russia) and 5% citrate buffer with added glucose (ACD-A). One milliliter of the resulting retroviral solution was added to each well of a 24-well plate pre-coated with 500 μL of PBS supplemented with 20 μg/mL retronectin (Takara Bio, Kusatsu, Japan) and centrifuged at 2000× *g* for 2 h at 32 °C. The wells were then washed twice with PBS containing 2% human serum albumin and seeded with 1.5 × 10^6^ T cells stimulated with mouse anti-CD3ε/-CD28 antibodies supplemented with 60 U/mL hIL-2 (Roncoleukin, Novosibirsk, Russia) and 10 ng/mL IL-7 (BioLegend, USA) in 1.5 mL full media RPMI-1640. The plates with the cells were centrifuged at 1000× *g* for 10 min at 32 °C. The cells were incubated for 4 days at 37 °C and 5% CO_2_. The viability of the cell cultures was determined by trypan blue staining and microscopic visualization. Transduction efficiency was assessed by flow cytometry and was 51.34 ± 2.94% (mean ± standard deviation, n = 6), see details in [24]. Transduction of mouse T lymphocytes was additionally confirmed by PCR (Supplementary S2). Total RNA was isolated using the Total RNA Purification Plus Kit according to the manufacturer’s instructions (Norgen Biotec, Thorold, Canada). The concentration and purity of the obtained RNA samples were assessed using a NanoDrop 2000 spectrophotometer (Thermo Fisher Scientific, Waltham, MA, USA). Expression of GD2-specific CAR was confirmed by PCR using the primers GCCCTCTTATGGCGTTCACT (forward) and AGTTGGTAATGCCTCCAGCC (reverse) (Supplementary S2). Amplification was performed using a CFX96 Touch Real-Time PCR Detection System (Bio-Rad Laboratories, Hercules, CA, USA).

### 4.7. Determination of GD2-Specific CAR-T Cell Subpopulations and GD2 Antigen Expression in the Melanoma Cell Line B78-21 by Flow Cytometry

CAR T cell subsets were assessed by flow cytometry. Cells at a concentration of 2 × 105 were stained using a cocktail of monoclonal antibodies: anti-CD3-Alexa Fluor^®^ 700, anti-CD4-Brilliant Violet 570TM, anti-CD8a-PE/Cyanine7, anti-CD44-APC/Cyanine7, and anti-CD62L-Alexa Fluor 488 (BioLegend, USA). Expression of the GD2 antigen by the B78-21 melanoma cell line was assessed using anti-GD2-PE (clone 14G2a) antibodies (BioLegend, USA). Samples were incubated for 30 min in the dark, washed with 1 mL of PBS solution containing NaN3, and analyzed on an Attune NxT flow cytometer (Thermo Fisher Scientific, USA).

### 4.8. Co-Cultivation of Tumor Cells and GD2-Specific CAR-T Cells

Transduced and non-transduced mouse T cells were co-cultured with B78-21 melanoma tumor cells for 24 h. Target cells were pre-seeded at 5 × 10^5^ per well and incubated overnight; then, the medium was completely replaced, and 5 × 10^6^ transduced or non-transduced cells were added at a tumor:effector ratio of 1:10. After 24 h of co-culture, the cells were collected and sorted using a MojoSort™ magnetic sorter. This method of magnetic sorting has been described in detail previously (see above).

### 4.9. Sample Preparation

The actual number of cells in each aliquot was re-verified using a microscope. Sample preparation and analysis of the samples were performed according to the protocol described in [32]. Then, the samples were centrifuged for 5 min at 1000× *g* to avoid cell damage. The culture medium was removed, and the cell pellet was resuspended in 1 mL of DPBS. This wash step was repeated twice under the same conditions. After the final wash, DPBS was removed, and 100 µL of Milli-Q water was added to the cell pellet. The sample was pipetted thoroughly to ensure complete resuspension. Samples were then frozen and stored at −80 °C until transported to the analytical laboratory. Before analysis, samples were thawed at room temperature and adjusted with Milli-Q water to final concentrations of 1,000,000 cells per 100 µL. Each sample was subjected to two freeze–thaw cycles (freezing at −70 °C and thawing at room temperature) to promote complete disruption of the membrane structure. Afterward, the samples were sonicated for 5 min. Then, 100 µL of the resulting lysate was transferred to a clean 1.5 mL tube and mixed with 400 µL of a cooled 1:1 methanol:acetonitrile mixture containing internal standards—3-quinuclidinol and 2-iodoadenosine—each at a final concentration of 250 ng/mL. The samples were vortexed on a thermoshaker for 20 min at 22 °C and 900 rpm, followed by centrifugation at 16,000× *g* for 15 min at 4 °C. The supernatant was transferred into a vial for LC-MS/MS analysis.

### 4.10. LC-MS/MS Analysis

The methanol and acetonitrile used for sample preparation were of HPLC gradient grade and were purchased from Chimmed (Moscow, Russia). The acetonitrile used for chromatographic separation was of LC-MS grade (purchased from Concord Technology, Tianjing, China). Purified water was obtained using a Sartorius Arium 611 DI system (Sartorius AG, Göttingen, Germany).

The samples were analyzed using high-performance liquid chromatography coupled with tandem mass spectrometry (LC-MS/MS) according to the method described in [33]. Chromatographic separation was performed on an LC-20AD Prominence system (Shimadzu, Japan) equipped with an SIL-20AC autosampler (Shimadzu, Kyoto, Japan) and thermostated at 10 °C. Eluent A consisted of 5% acetonitrile in an aqueous solution of 20 mM (NH_4_)_2_CO_3_, adjusted to pH 9.8 with aqueous ammonia. Eluent B was 100% acetonitrile. Each sample was analyzed in duplicate, namely, under hydrophilic interaction liquid chromatography (HILIC) and reversed-phase chromatography (RP LC) modes. The HILIC gradient was as follows: 0 min—98% B, 2 min—98% B, 6 min—0% B, and 10 min—0% B. The column was then re-equilibrated for 4 min. The RP LC gradient was as follows: 0 min—0% B, 1 min—0% B, 6 min—98% B, and 16 min—98% B. The column was then re-equilibrated for 3 min. The flow rate for both methods was 300 µL/min. The injection volume was 10 µL. Both chromatographic modes were conducted using a monolithic column based on 1-vinyl-1,2,4-triazole, 2 × 60 mm. The monolithic column material was synthesized according to the procedure described in [34]: copolymerization was carried out in a glass tube with an internal diameter of 2 mm using a monomer mixture of styrene/divinylbenzene/1-vinyl-1,2,4-triazole taken at a volume ratio of 10:50:40, respectively.

Mass spectrometric detection of 489 metabolites (358 analyzed in HILIC mode and 131 in RP LC mode) was performed in multiple reaction monitoring (MRM) mode, using both positive and negative ionization, on an API 6500 QTRAP mass spectrometer (AB SCIEX, USA) equipped with an electrospray ionization (ESI) source. The main mass spectrometric parameters were as follows: ion spray voltage (IS) was 5500 V and –4500 V for positive and negative ionization modes, respectively; desolvation gas temperature (TEM) was 475 °C; collision gas (CAD) set to Medium; and nebulizer gas (GS1), auxiliary gas (GS2), and curtain gas (CUR) pressures were 33, 33, and 30 psi, respectively. The declustering potential (DP) was ±91 V, the entrance potential (EP) was ±10 V, and the collision cell exit potential (CXP) was ±9 V. The dwell time for each MRM transition was 5 ms. Precursor and fragment ion transitions, metabolite names, fragmentation times, and corresponding collision energies were adapted from [35,36]. Instrument control and data acquisition were performed using Analyst 1.6.3 software (AB SCIEX, Marlborough, MA, USA). Chromatograms were processed using Skyline [37] software (Skyline version 24.1, https://skyline.gs.washington.edu accessed on 15 November 2025).

### 4.11. LC-MS/MS Data Analysis

We analyzed the obtained peak area data as a .csv file using our own Python 3 code via Jupyter Notebooks 4.3.4 version. To plot the PCA plot, heatmap, and correlation matrix, we used the following code (https://github.com/Perik-Zavodskii/BulkOmicsTools/blob/main/BulkOmicsTools.ipynb accessed on 20 December 2025). Based on this code, we modified the data, performed normalization, statistical processing, and created a PLS-DA plot.

### 4.12. Production of Spheroids of the B78-21 Line Melanoma

To prepare the required medium for 3D-culture formation using spheroids, we used RPMI culture medium with 0.5% carboxymethylcellulose (CMC) content. 3D culture of B78-21 was obtained using the Hanging drop method. Briefly, cells were plated on a Petri dish drop lid with a concentration of 1 × 10^4^ cells per 25 µL volume; after that, 25 mL of PBS was added to the Petri dish, gently covered with a Petri dish drop lid, and placed in a CO_2_ incubator for 7 days. Then, after 7 days, the lid was removed from the cup, and the formed spheroids were gently washed from top to bottom into the bottom of the lid. Each spheroid was collected with a spout and scraped into wells, and medium was added at a concentration of 1 spheroid per 200 µL volume. For the next 4 days, spheroids were cultured to optimum size (1 mm), changing the medium every 48 h. The obtained spheroids were evaluated for the formation of proliferation and quiescent zones. In short, the spheroids were stained with supra-vital dyes Calcein for the detection of live cells and Rhodamine 123 for the detection of dead cells.

### 4.13. Extracellular Acidification Rate (ECAR) and Basal Oxygen Consumption Rate (OCR) Measured by a Seahorse XFp Analyzer

To analyze GD2-specific CAR-T cell metabolism using a Seahorse XFp analyzer, cells were co-cultured at a ratio of 2 spheroids per 4 × 10^5^ CAR-T cells for 24 h. After co-culture, cells were sorted using a MojoSort™ Mouse CD3 T Cell Isolation Kit (Biolegend, San-Diegom CA, USA). For analysis, 2.5 × 10^5^ cells were taken on Cell Culture Miniplates (Seahorse XFp FluxPak, 103022-100, Agilent Technologies, Santa Clara, CA, USA). To ensure cell adherence, Poly-L-lysine (PanEco, Moscow, Russia) was applied to the miniplates at a concentration of 22.4 ng/mL; seeded adherent cells were placed in a CO_2_ incubator overnight. At the same time, 200 µL of distilled water per well was added to the plate. Meanwhile, for the cartridge sensors (Seahorse XFp FluxPak, 103022-100, Agilent Technologies, Santa Clara, CA, USA), 200 µL ddH2O was added to each well for hydration, overnight, in a 37 °C non-CO_2_ incubator. The next day, the water was replaced with XF Calibrant (100840-100, Agilent Technologies), and cartridge sensors were immersed in the XF Calibrant and incubated for 1 h in a 37 °C non-CO_2_ incubator. Seeded adherent cells were removed from the incubator and washed 1 time in XF media (nonbuffered DMEM containing 25 mM glucose, 2 mM L-glutamine, and 1 mM sodium pyruvate), and then incubated for 1 h in a 37 °C non-CO_2_ incubator. After incubation, Cell Culture Miniplates with adherent cells were added up to 180 µL XF media. Oxygen consumption rates (OCR) and extracellular acidification rates (ECAR) were analyzed in XF media under basal conditions and in response to 1 µM oligomycin (port A) and 0.5 µM rotenone with antimycin A (port B) (Seahorse XF Real-Time ATP Rate Assay Kit, 103591-100, Agilent Technologies). The utility plate filled with XF Calibrant and capped with the cartridge is positioned in the Seahorse XFp Analyzer tray (Agilent Technologies), and the calibration of the signals generated by all 8 wells is performed. The culture plate is then introduced into the tray, and the acquisition program is run. This program includes a step of equilibration followed by basal, post-oligomycin injection, and post-Rotenone/Antimycin A injection measurements. Each of these three steps comprises three loops of three minutes mixing, two minutes waiting, and three minutes measuring. The raw data were statistically processed using Seahorse Wave 2.6.3 software (Agilent Technologies) and then presented graphically using Graphpad Prism version 10.0.1.

### 4.14. Measure Δψm and ROS by in Cell Analyzer 6000

To assess the metabolic activity of GD2-specific CAR-T cells during culturing with 3D culture-derived B78-21 tumor cells, fluorescence microscopy was performed using In Cell Analyzer 6000 with Cell Culture Life Support Module (GE Healthcare, USA). The metabolic activity of GD2-specific CAR-T cells was assessed by evaluating the membrane potential of cell mitochondria (Δψm) and the production of reactive oxygen species (ROS). For this purpose, one day before co-culturing, GD2-specific CAR-T cells were cultured in RPMI-1640 medium supplemented with Tetramethylrhodamine (TMRM) to assess Δψm and CEllROX to assess ROS, and 100 μg of Verapamil was added to inhibit Na/Ca channels to avoid dye export from the cytoplasm of GD2-specific CAR-T cells. On the day of analysis, the labeled GD2-specific CAR-T cells were co-cultured with a 3D culture of tumor cell line B78-21 spheroids prepared in a 96-well plate (Eppendorf, Framingham, MA, USA). Microscopic images in light-field, dsRed (TMRM), and Cy5 (CEllRox) channels were taken at 20× resolution every 2 h until 24 h after cell seeding. For each well, 9 fields of view in each well of the plate were analyzed. Counting of detected cells at each time point was performed using In Cell Developer Toolbox 1.9.3 software (GE Healthcare, Chicago, IL, USA).

### 4.15. Statistical Analysis

Statistical processing of data was performed using GraphPad Prism 9.4.1 software (GraphPad Software, Boston, MA, USA). The obtained data were tested for normality by the Kolmogorov–Smirnov and Shapiro–Wilk tests. The data were further analyzed using suitable statistical methods. The statistical methods (including the statistical test used, exact value of n, and what n represents) used are indicated in the figure captions.

## 5. Conclusions

The study of the interplay between metabolic processes and immune function in T-lymphocytes is a pressing issue today. Combined approaches to studying the impact of metabolism on the immune cells’ effector function offer valuable insights into the interactions between T lymphocytes and tumor cells of various lineages. This knowledge will improve the effectiveness of adoptive cell therapy against tumors and reduce the cytotoxic effect of modified T lymphocytes on patient tissue. Therefore, studying metabolism provides an additional opportunity to implement new approaches to treating various diseases.

## Data Availability

The original contributions presented in this study are included in the article/Supplementary Materials. Further inquiries can be directed to the corresponding author(s).

## Supplementary Materials

The following supporting information can be downloaded at: https://www.mdpi.com/article/10.3390/ijms27115093/s1.
