# Supplementary figures and images for: Changes in the Metabolism of GD2-Specific Murine CAR-T Cells After Co-Culturing with Melanoma
Source: Int J Mol Sci. 2026 Jun 4;27(11):5093. doi: 10.3390/ijms27115093 (PMC13256618; doi:10.3390/ijms27115093)

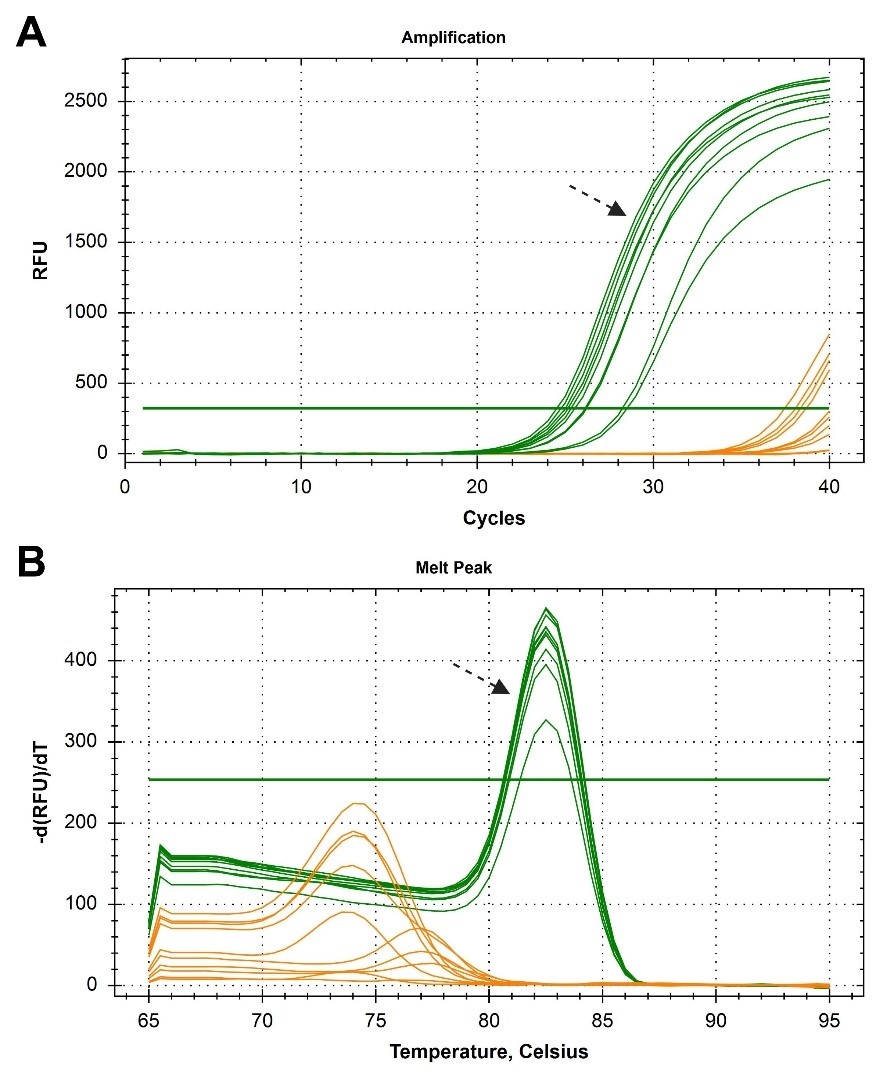

Supplement: Supplementary file 1 [file ijms-27-05093-s001.zip › ijms-4316293-supplementary-s2.jpg]
